# Pollen adaptation to ant pollination: a case study from the Proteaceae

**DOI:** 10.1093/aob/mcaa058

**Published:** 2020-03-28

**Authors:** Nicola Delnevo, Eddie J van Etten, Nicola Clemente, Luna Fogu, Evelina Pavarani, Margaret Byrne, William D Stock

**Affiliations:** 1 Centre for Ecosystem Management, Edith Cowan University, Joondalup, WA, Australia; 2 Department of Chemistry, Life Sciences and Environmental Sustainability, University of Parma, Parma, Italy; 3 Biodiversity and Conservation Science, Department of Biodiversity, Conservation and Attractions, Bentley Delivery Centre, Bentley, WA, Australia

**Keywords:** Australia, ant–plant interaction, biodiversity hotspot, *Conospermum undulatum*, cuticular antimicrobial secretions, entomophily, floral fidelity, Hymenoptera, myrmecophily, mutualism, pollen germination

## Abstract

**Background and Aims:**

Ant–plant associations are widely diverse and distributed throughout the world, leading to antagonistic and/or mutualistic interactions. Ant pollination is a rare mutualistic association and reports of ants as effective pollinators are limited to a few studies. *Conospermum* (Proteaceae) is an insect-pollinated genus well represented in the south-western Australia biodiversity hotspot, and here we aimed to evaluate the role of ants as pollinators of *C. undulatum*.

**Methods:**

Pollen germination after contact with several species of ants and bees was tested for *C. undulatum* and five co-flowering species for comparison. We then sampled the pollen load of floral visitors of *C. undulatum* to assess whether ants carried a pollen load sufficient to enable pollination. Lastly, we performed exclusion treatments to assess the relative effect of flying- and non-flying-invertebrate floral visitors on the reproduction of *C. undulatum*. For this, we measured the seed set under different conditions: ants exclusion, flying-insects exclusion and control.

**Key Results:**

Pollen of *C. undulatum*, along with the other *Conospermum* species, had a germination rate after contact with ants of ~80 % which did not differ from the effect of bees; in contrast, the other plant species tested showed a drop in the germination rate to ~10 % following ant treatments. Although ants were generalist visitors, they carried a pollen load with 68–86 % of suitable grains. Moreover, ants significantly contributed to the seed set of *C. undulatum*.

**Conclusions:**

Our study highlights the complexity of ant–flower interactions and suggests that generalizations neglecting the importance of ants as pollinators cannot be made. *Conospermum undulatum* has evolved pollen with resistance to the negative effect of ant secretions on pollen grains, with ants providing effective pollination services to this threatened species.

## INTRODUCTION

Mutualistic plant–animal interactions are a common ecological process with almost 90 % of wild flowering plant species relying on animals for gamete dispersal and, ultimately, fruit and seed production (Ollerton *et al.*, 2011). Most animals involved in such interactions are insects, and they account for the pollination of ~88 % of all animal-pollinated plants (Potts *et al.*, 2010; Thomann *et al.*, 2013). Among the insect-pollinated plants, pollination by ants appears to be poorly represented (de Vega and Gómez, 2014; Kuriakose *et al.*, 2018; Rostás *et al.*, 2018; Del-Claro *et al.*, 2019), whereas bees and other close relatives are recognized as important pollinators worldwide (Potts *et al.*, 2016). Moreover, interactions between ants and flowers are generally assumed to be antagonistic. This large discrepancy between the recognized roles of bees and ants has been attributed to peculiar characteristics of ants, such as their small size (being generally smaller than the reproductive structures of flowers), their aggressive behaviour that may deter other flower visitors, and their grooming, or self-cleaning, behaviour (Galen, 1983; Junker *et al.*, 2007). Ants are also known to produce an antimicrobial secretion from their metapleural gland, which has been shown to have a negative effect on the viability of pollen (Beattie *et al.*, 1985). This trait may have contributed to differences in pollination efficacy among the major hymenopteran lineages (i.e. the ‘antibiotic hypothesis’; Beattie *et al*., 1984, 1985). The primary function of this cuticular secretion is very likely antiseptic (Poulsen *et al.*, 2002; Stow and Beattie, 2008; Yek and Mueller, 2011), with ants spreading antibiotic secretions diffusely through the nest to prevent fungal growth and infections (Hölldobler and Wilson, 1990). Possibly, this is the reason why ant pollination appears to be mainly limited to dry, or sometimes cold, environments (Dutton and Frederickson, 2012); indeed, bacteria and fungi are likely to impose stronger selection on ants for antimicrobial defences in warm, humid tropical rainforests than in deserts and Mediterranean-type habitats. Nonetheless, ant pollination may be an advantageous system with a low energetic cost, and could be favoured in habitats where ant frequency is high and plants produce small, open flowers with low amounts of pollen (i.e. the ant-pollination syndrome; Hickman, 1974). Reports of ants as effective pollinators are limited to a low number of convincing examples (46) (de Vega and Gómez, 2014) with the number of such studies increasing over recent years (Domingos-Melo *et al.*, 2017; Del-Claro *et al.*, 2019) suggesting that further studies are needed to evaluate some of the earlier generalizations about the negative role of ants as pollinators.

Ants are known to play an important role in seed dispersal in a number of regions and ecosystems (Lengyel *et al.*, 2010; Suetsugu *et al.*, 2017; Luna *et al.*, 2018; Magalhães *et al.*, 2018), including the sandplains of south-west Australia (also known as ‘kwongan’). The region is noted for its rich floral diversity, especially among the medium-sized shrubs of the families Proteaceae, Myrtaceae and Ericaceae (Hopper and Gioia, 2004). It is characterized by an old, stable landscape and nutrient-poor soil (Hopper, 1979) with a climate that is typically Mediterranean with most rain concentrated in the winter months.

Despite many theories that have advanced the importance of ant dispersal (Majer, 1982; Gove *et al.*, 2007), little attention has been given to their possible role as pollinators in these regions. This became apparent during our recent studies on the pollination ecology of a threatened member of the Proteaceae (*Conospermum undulatum*) where we observed that ants were the second-most active floral visitors for this species (N. Delnevo *et al*., unpubl. res.). Thus, *C. undulatum* could represent a potential model species to test for ant pollination in a region where ants are abundant and diverse, and are already well known for their ecological role in dispersing seeds from many plant species, including members of the Proteaceae.

In this study, we evaluate the effectiveness of ants as pollinators and whether they negatively interfere with plant reproduction by rendering pollen grains unviable (and thus robbing nectar from the flowers) by assessing the effect of ant secretions on pollen germination. A lack of a negative response to ants could result from either the low production of secretions by local ants or because a plant species has adapted to potentially use ants as pollen vectors by producing pollen resistant to secretions. Therefore, to test for potential local adaptation we compared the response to ant secretions across several species of native ants and species of the Proteaceae. Possible reduced selection for antimicrobial secretions in this dry Mediterranean-climate region and observations of ants visiting flowers suggest ants may act as effective pollinators in the region. On the other hand, ants may still produce antimicrobial secretions, but some plant species may have adapted to cope with such secretions, although this has never been tested before.

The effectiveness of a given pollinator not only depends on its floral visitation but also on the efficiency with which they deposit conspecific pollen (Herrera, 1987). Ants commonly are generalist floral visitors; however, short-term pollinator foraging specialization on a particular plant species, known as floral fidelity, may occur (Brosi, 2016). For most plants, floral fidelity is critical because transfer of conspecific pollen must occur in order for fertilization to take place, so we investigated whether ants carry a suitable conspecific pollen load to enable successful pollination in *C. undulatum*. We also carried out an exclusion experiment to demonstrate if ants are effective pollinators in *C. undulatum* and to evaluate to what extent ants contributed to the reproductive output of this species. We hypothesized that because of the generally restricted foraging range of ants in comparison to winged hymenopterans and their possible antibiotic production, their contribution to seed set would be expected to be negligible (or negative) relative to naturally pollinated plants and those pollinated by flying insects, which we expected to be similarly high.

## MATERIALS AND METHODS

### Study area and species

The study was conducted in south-west Western Australia within the Swan Coastal Plain bioregion. This region is a low-lying coastal plain that extends from Jurien Bay, north of Perth, to Cape Naturaliste in the south, and it is part of the Southwest Australia global biodiversity hotspot (Mittermeier *et al.*, 2004). The area experiences a dry, Mediterranean-type climate (Beard, 1984), with hot dry summers (December–March), and mild wet winters (June–August) with 600–1000 mm of rainfall on average across the region. The area is characterized by deep, highly leached sand dune systems (McArthur and Bettenay, 1974) with low woodland dominated by *Banksia* trees and highly diverse shrubby understorey.

*Conospermum* (Proteaceae) is an insect-pollinated genus endemic to Australia with its centre of distribution being the south-west corner of Western Australia. The genus includes 53 species (Bennett, 1995) and is of increasing conservation concern, with four taxa already listed among the threatened flora of Western Australia (Government Gazette of Western Australia, 2018). Like all Proteaceae, the perianth has four tepals, although in *Conospermum* the tepals are of unequal size, with the upper one being much larger than the other three. Zygomorphy is expressed in the bilabiate perianth, the upper tepal forming a broad hood over the other three tepals, in each of which the distal-most portion flares and reflexes downward, allowing entry to the flower (Bennett, 1995; Douglas, 1997). The flowers of *Conospermum* possess an active pollination mechanism. The style is bent, and the flower opens in a state of tension [Stone *et al.*, 2006; but see Douglas (1997) for a morphological description]. When a visiting insect applies pressure with its mouthparts at the base of the style it flicks away from the fertile anthers and strikes the visitor. The moist cup-shaped stigma is forced down onto the pollinator and thereby picks up pollen carried by the insect; at the same time the fertile anthers dehisce explosively, casting new pollen onto the visitor (Morrison *et al.*, 1994; Stone *et al.*, 2006). Thus, *Conospermum* flowers need to be visited by insects carrying a suitable pollen load from previous floral visits in order for pollination to occur, leading to development of fruits. These are cone-shaped, covered with tan orange hairs, and contain only one seed (i.e. achenes).

**Fig. 1.**
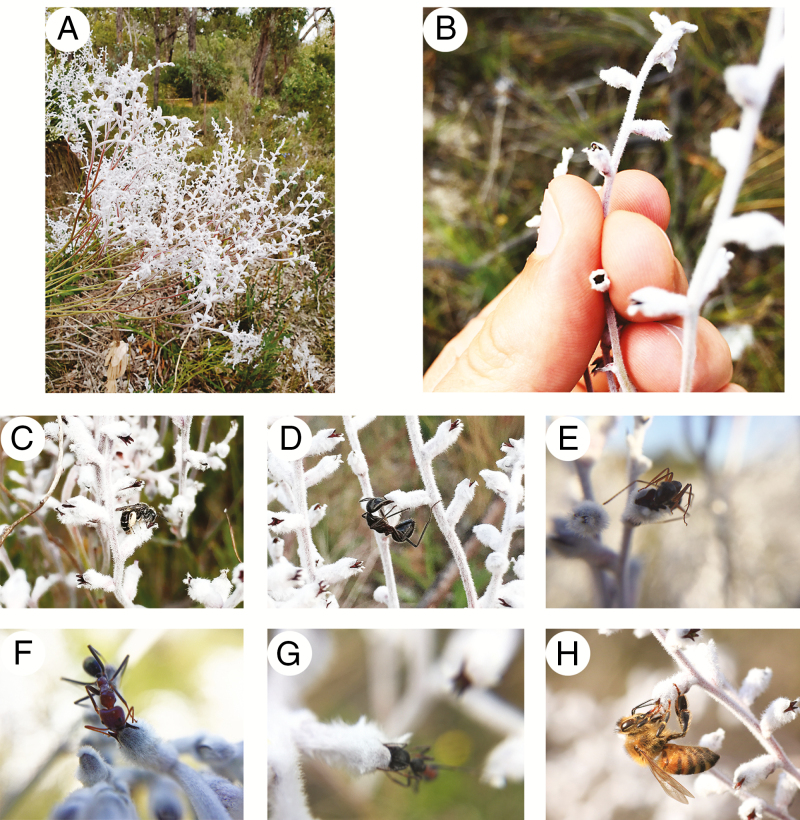
(A) White flowers of *Conospermum undulatum* stand out in the understorey of *Banksia* woodland. (B) Detail of flowers of *C. undulatum*. (C–H) Insects visiting flowers of *C. undulatum*: (C) *Leioproctus conospermi*; (D) *Camponotus molossus*; (E) *Camponotus terebrans*; (F) *Iridomyrmex purpureus*; (G) *Myrmecia infima*; (H) *Apis mellifera*. Note that *A. mellifera* only insert its proboscis into the flower to steal nectar.

In particular, *Conospermum undulatum* is a monoecious plant that grows as an erect, compact shrub up to 1.5 m tall with distinctive fibrous, longitudinally fissured stems. The glabrous leaves are to 12 cm long and 3.8 cm wide with a characteristic undulating margin. This species is currently listed in the threatened flora of Western Australia (Government Gazette of Western Australia, 2018) and has been assessed as ‘Vulnerable’ using IUCN red list criteria (Department of Environment & Conservation, 2009). It was originally considered a variety of *C. triplinervium*, which also occurs in the region but with different habit and leaf morphology (Bennett, 1995). Molecular evidence has established *C. undulatum* as a distinct species (Close *et al.*, 2006), and recently developed genetic resources are being used to further clarify genetic relationships among populations (Delnevo *et al.*, 2019*a*). The flowering period usually ranges from late August to late October. In a recent study, Delnevo *et al.* (2019*b*) found that the pollination mechanism in *C. undulatum* is an effective physical barrier against autogamous selfing, and also found that this species possesses a strongly developed self-incompatibility system that prevents the development of the embryo following geitonogamous selfing. The hermaphroditic flowers are small, measuring ~7 mm in length, with the tube being ~4 mm. They are covered in white hairs and are produced in inflorescences held well above the leaves. Flowers do not produce any obvious scent and offer a nectar reward located within the flower, at the base of the calyx tube. In this way an insect would trigger the mechanism by pushing on the trigger point near the anthers with its mouthparts whilst scavenging for nectar and/or pollen (Fig. 1).

Due to its characteristic floral morphology and pollination system, *C. undulatum* relies on a restricted group of pollinators, mainly hymenopterans. The native bee *Leioproctus conospermi* (Colletidae) and native ants, including sugar ants, meat ants and bull ants, are the most active floral visitors of this species (N. Delnevo *et al*., unpubl. res.).

### Pollen germination assays

To determine local adaptations of plants to cope with the detrimental effect of ant secretions on pollen viability, we performed a pollen germination assay to compare the germination of pollen collected from *C. undulatum* to that of five other plant species after contact with three species of Australian ants, as well as honeybees and a control (no contact with insects). Specifically, we selected the following ant species: *Iridomyrmex purpureus*, found throughout Australia, including our study region; *Camponotus terebrans*, mainly found in the southern part of Australia; and *Camponotus molossus*, native to the Swan Coastal Plain (Heterick, 2009). Following several field surveys we were unable to find any nests of the bull ant *Myrmecia infima*, so we were unable to test the response of pollen with this ant species even though it was observed visiting *Conospermum* flowers.

The plant species selected for this experiment were *Conospermum undulatum*, *Conospermum stoechadis*, *Conospermum canaliculatum*, *Grevillea eriostachya*, *Grevillea leucopteris* and *Banksia nivea*. These species were selected as they are co-flowering shrub species that co-occur in the Swan Coastal Plain, and all three species of ant were recorded visiting flowers of these plants. They all belong to the family Proteaceae and were collected within 20 km of the centre of the distribution of *C. undulatum*. For each species, freshly opened flowers within 1 d of anthesis were collected on the same morning they were used. In the laboratory we pooled pollen from several flowers of the same species in Petri dishes. Subsequently, we gently picked up each ant or bee with tweezers, lightly dabbed it in the pollen grains and put the live insect in a clean 50-mL centrifuge tube for 30 min, a standard exposure time used in several similar studies (e.g. Peakall and Beattie, 1989; Dutton and Frederickson, 2012). For the control we left pollen grains in an empty tube for the same amount of time. Next, we transferred the pollen from ants, bees or controls onto a microscope slide with a drop of pollen germination medium by gently dipping the insect into the drop and placed a coverslip to prevent desiccation. The pollen germination medium was prepared following a modified version of Brewbaker and Kwack (1963); briefly, the medium was made up of 100 mg L^−1^ of boric acid, 300 mg L^−1^ calcium nitrate, 200 mg L^−1^ magnesium sulphate, 100 mg L^−1^ potassium nitrate and 20 % sucrose. The selected concentration of sucrose was found to be the one that maximized pollen tube growth for all the tested species following trials ranging from 10 to 60 % sucrose. After an incubation period of 48 h in the dark at room temperature (24 °C) we assessed the germination rate by counting the number of pollen grains with and without pollen tubes under a microscope. We tested pollen from each plant species against five individual workers of each ant species, five individual worker bees and five controls (*n* = 150 germination assays).

### Floral fidelity

In the field, we sampled the pollen load of ten individuals of each species of floral visitor of *C. undulatum*. The insects were collected from inflorescences of *C. undulatum* using clear 50-mL centrifuge tubes after recording whether there was stigmatic contact. To avoid contamination of the pollen load a clean tube was used for every insect. We induced cold anaesthesia by placing the tube containing the insect on ice, and removed pollen non-destructively by dabbing the pollinator body in a standardized manner (i.e. two dabs on the head and forehead) with a cube of fuchsin-stained gel (Kearns and Inouye, 1993; Brosi and Briggs, 2013). The captured insect was released as soon as the pollen had been sampled. We then mounted the pollen-containing gel on microscope slides and assessed floral fidelity in each pollen load by sorting pollen grains as either ‘*C. undulatum*’ or ‘other species’ by means of a pollen reference slide of *C. undulatum*. To account for possible contamination in the field, we classified pollen loads as monospecific if >95 % of pollen grains represented *C. undulatum*, and as heterospecific if otherwise, following the approach of Brosi and Briggs (2013).

### Exclusion experiment

Autogamous selfing and anemophily have already been tested recently by Delnevo *et al.* (2019b) and no fruits were recorded in these total exclusion treatments, demonstrating that *C. undulatum* relies completely on pollinators for pollen transfer. In this study, we aimed to experimentally assess the relative contribution of ants and flying visitors to the reproductive output of *C. undulatum*. We performed three experimental treatments in the field: flying insect exclusion (FLY_EXC), ant exclusion (ANT_EXC) and control (flowers freely exposed to all visitors). In three contiguous patches of *C. undulatum* characterized by similar population size (between 400 and 600 plants) we randomly selected a total of 27 plants. To implement the FLY_EXC treatment we covered the selected plants 1 week prior to anthesis with a net tent (0.25-mm^2^ mesh) to 2 cm from the ground, so that only crawling insects could visit the inflorescences. Net tents were monitored for the presence of flying insects every week for the entire flowering period to ensure their efficacy, and no flying insects were recorded. The ANT_EXC treatment was performed by applying Tanglefoot around the woody stems of selected *C. undulatum* plants 1 week prior to anthesis, to prevent crawling insects from reaching the opened flowers. At the end of the flowering period, when flowers began to senesce, we placed fine mesh bags around the inflorescences to collect the fruits. In the laboratory, we counted the number of flowers, fruits and seeds collected for each plant. The number of flowers was assessed by counting the scars left on the white, woolly inflorescence stalk of *C. undulatum*, and we obtained a total of 3935 flowers.

### Data analysis

Data from the germination assays were analysed using a generalized linear model (GLM) with the proportion of germinated pollen as the response variable and plant species, treatment and their interaction as the explanatory variables. We then compared all the combinations of levels of the explanatory variables with a Tukey’s HSD test.

To analyse whether visitors showed floral fidelity, or they were generalists, we fitted a generalized linear mixed effect model (GLMM) with the proportion of *C. undulatum* pollen within the pollen load as the response variable, and the visitor taxon as the explanatory variable. Because individuals of the insect and plant species studied within a study site are likely to be closely related genetically, and environmental conditions are similar, data collected within a study site are not independent. To address this lack of independence and prevent pseudoreplication, we used *Conospermum* population as a random effect. Again, we compared each level of the explanatory variable with a Tukey’s HSD test. Syrphid flies (Syrphidae) were excluded from the analysis because of extremely small pollen load, whereas *Myrmecia infima* was excluded because we were unable to collect enough pollen load from this species in the field.

Finally, we used the proportion of seeds out of the total number of flowers as the response variable in a GLMM with the exclusion treatments as the explanatory variable and *Conospermum* patch as the random effect.

All of our response variables were proportions, and therefore we used binomial error distribution (appropriate for proportional data) to account for non-normal distribution of residuals and non-homogeneous variances in each model, and checked that the assumptions were fulfilled by visual inspection of residual patterns (Zuur *et al.*, 2009). All statistical analyses were performed with R version 3.5.2 (R Development Core Team, 2018).

## RESULTS

### Pollen germination assays

**Fig. 2.**
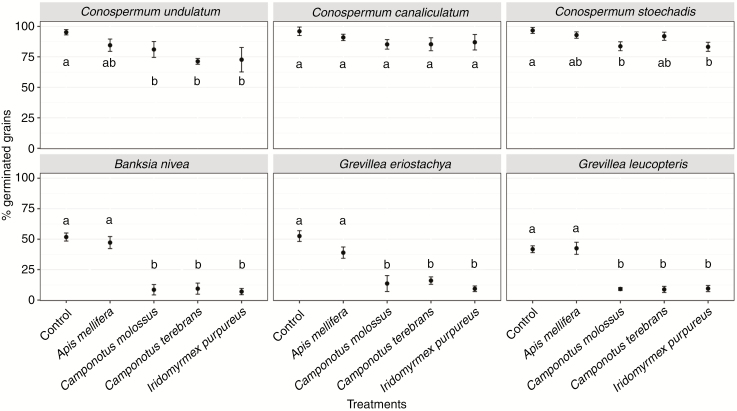
Pollen grain germination assays of six plant species. Mean (% ± s.e.) pollen germination after contact with the different treatments; treatments marked by different letters are significantly different at α = 0.05 according to Tukey’s HSD tests.

The pollen germination response was different among treatments and the significant interaction term indicates different responses to the same treatment among species (Table 1). Pollen of *Conospermum* species subject to the control treatment had the highest germination response, with *C. undulatum*, *C. stoechadis* and *C. canaliculatum* having 95.2, 96.7 and 96 % of pollen grains germinated after the incubation period of 48 h, respectively (Fig. 2). The germination rates of pollen from the other plant species subject to the control treatment were all lower than that of *Conospermum*, and had similar germination rates of ~50 %, with the least responsive species being *G. leucopteris* (41.8 %).

**Table 1.** Analysis of variance table showing the effects of plant species, treatments and their interactions on pollen germination response

Variable	d.f.	χ ^2^	*P*
Plant species	3	511.37	<0.001
Treatment	4	360.03	<0.001
Plant species ×Treatment	12	21.35	0.04

**Fig. 3.**
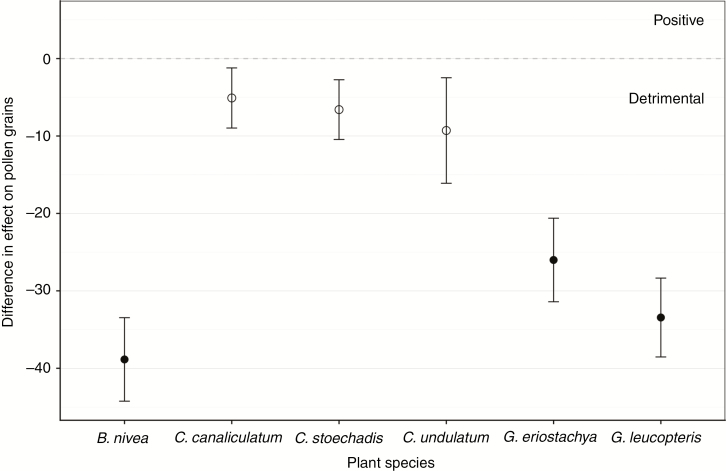
Difference between the effect of ants (pooled together) and the effect of *A. mellifera* on pollen germination; dots below the dashed line indicate a negative effect of ants. Closed dots indicate a statistically significant difference between *A. mellifera* and ants, open dots no significant difference.

The test of the effect of honeybees showed there was no significant detrimental effect on pollen germination after contact with *A. mellifera* in any tested plant species compared to control treatments (Fig. 2). In contrast, contact with ants severely reduced the pollen germination to ~10 % in *B. nivea*, *G. eriostachya* and *G. leucopteris*, but not in *Conospermum* species. In particular, *C. undulatum* had a pollen germination after contact with the integument (outer covering) of *Camponotus molossus*, *Camponotus terebrans* and *I. purpureus* of 81.1, 71.3 and 72.7 %, respectively. The germination rate in *C. stoechadis* and *C. canaliculatum* was similar to *C. undulatum*, and did not differ statistically from the effect of bees (Fig. 2). For *B. nivea*, *G. eriostachya* and *G. leucopteris*, contact with all the ant species led to significantly reduced pollen germination, being 38.9, 26 and 33.4 % lower respectively, compared to bees (*P* < 0.001 in all cases). In contrast, pollen germination in *C. undulatum*, *C. stoechadis* and *C. canaliculatum* was reduced by only 9.3, 6.6 and 5.1 % with ant exposure, respectively, and did not differ from the effect of bees (*P* = 0.532, *P* = 0.350 and *P* = 0.702; Fig. 3).

### Floral fidelity

**Fig. 4.**
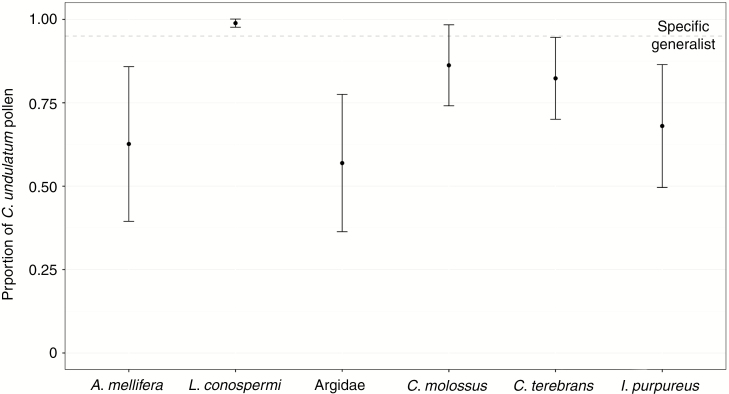
Proportion of *Conospermum undulatum* pollen grains (± s.e.) within the pollen load of insects recorded on *C. undulatum* plants. Dots above the dashed line represent insects that carried monospecific pollen load; dots below the line represent heterospecific pollen loads.

The native bee *Leioproctus conospermi* was the only species that carried monospecific pollen (mean ± SE = 0.989 ± 0.006; Fig. 4), which was significantly different from all other species of pollinators, indicating highly specialized pollination of *C. undulatum* (Table 2). Argid sawflies, *A. mellifera* and *I. purpureus* were the most generalist pollinators, carrying a pollen load with average proportions of *C. undulatum* pollen being 0.57, 0.63 and 0.68 respectively. The two *Camponotus* species showed high proportions of *C. undulatum* pollen grains within their pollen load, although these were not statistically different from the other generalist pollinators. In particular, *Camponotus terebrans* carried a pollen load with an average proportion of 0.82 of suitable grains, whereas *Camponotus molossus* had 0.86 (Fig. 4).

**Table 2.** Tukey HSD pairwise comparison of the floral fidelity of the different recognizable taxonomic units of visitors of flowers of *Conospermum undulatum*. Estimate of contrasts, s.e. and *P*-values are reported (***P < 0.001, **P < 0.01, *P < 0.05).

Contrast	Estimate	SE	*P*
Argidae *– A. mellifera*	−0.238	0.6234	0.999
*C. molossus – A. mellifera*	1.318	0.6641	0.349
*C. terebrans – A. mellifera*	1.021	0.6096	0.545
*I. purpureus – A. mellifera*	0.238	0.6106	0.999
*L. conospermi – A. mellifera*	3.963	0.7212	<0.001***
*C. molossus –* Argidae	1.556	0.6186	0.118
*C. terebrans* – Argidae	1.260	0.5597	0.212
*I. purpureus –* Argidae	0.477	0.5607	0.957
*L. conospermi* – Argidae	4.202	0.6795	<0.001***
*C. terebrans – C. molossus*	−0.296	0.6047	0.997
*I. purpureus – C. molossus*	−1.079	0.6057	0.475
*L. conospermi – C. molossus*	2.645	0.717	0.003**
*I. purpureus – C. terebrans*	−0.783	0.5454	0.703
*L. conospermi – C. terebrans*	2.942	0.6669	<0.001***
*L. conospermi – I. purpureus*	3.725	0.6678	<0.001***

*A. mellifera*, *Apis mellifera*; *C. molossus*, *Camponotus molossus*; *C. terebrans*, *Camponotus terebrans*; *I. purpureus*, *Iridomyrmex purpureus*; *L. conospermi*, *Leioproctus conospermi.*

### Field exclusion experiment

**Fig. 5.**
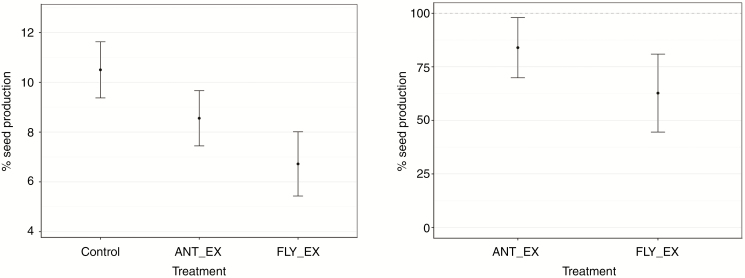
Seed production in *Conospermum undulatum* subject to experimental treatment. (A) Percentage of seeds produced by *C. undulatum* plants subject to treatments of natural pollination, ant exclusion and flying-visitor exclusion. (B) Relative seed set of *C. undulatum* plants subject to ant exclusion and flying-visitor exclusion compared to freely exposed natural pollinated plants; the dashed line indicates seed production of controls.

The probability that a flower developed a seed in freely exposed control plants was 10.5 %, whereas flowers available only to flying visitors (ANT_EX treatment) resulted in a probability of 8.6 % of seed set (Fig. 5A). Flying-visitor exclusion treatments (FLY_EX) showed that ants were effecting pollination, resulting in a probability of setting seed of 6.7 %. Using the controls as the reference for the maximum amount of seed that can be developed by freely exposed *C. undulatum* plants (Fig. 5B), the results showed that flying insects alone produced significantly fewer seeds than controls (84 %; *P* = 0.043), and that ants alone contributed to 62.7 % of the seed set of freely exposed control plants (*P* ≤ 0.001). The results of the two treatments ANT_EX and FLY_EX were not significantly different from each other (*P* = 0.096).

## DISCUSSION

Pollination is a critical element of plant sexual reproduction and our study within a key genus of Proteaceae has revealed that ants are important secondary pollinators for *C. undulatum*, a threatened species in the Australian kwongan. We found evidence that within the genus *Conospermum* plants have adapted the biochemistry of their pollen grains to favour the action of these secondary pollinators. In addition, we demonstrated that *C. undulatum* has a highly specialized pollination mutualism with a native *Leioproctus* bee. Identification of such specific pollination associations are important for management of threatened species to ensure maintenance of effective pollination services to ensure long-term population viability.

In contrast to the expectation under the antibiotic hypothesis where ant secretions mostly prevent the transfer of viable pollen (Beattie *et al*., 1984, 1985; but see Peakall and Beattie, 1989; Gómez and Zamora, 1992; Gómez *et al.*, 1996), we found that the germination of pollen grains was not inhibited in *C. undulatum*, or in the other species of this genus studied. The germination of pollen grains in *B. nivea*, *G. eriostachya* and *G. leucopteris*, on the other hand, was drastically reduced after contact with the ant treatment and is consistent with the antibiotic hypothesis and with observations in other temperate and tropical plant species where the pollen germination rate decreased after contact with several different species of ants (Dutton and Frederickson, 2012). The opposite outcomes between *Conospermum* and the other species suggest strongly that within the genus *Conospermum* plants have evolved to favour the action of ants as secondary pollinators by producing pollen with resistance to the negative effect of ant secretions on pollen grains that is common in the majority of plants. Moreover, the strong negative effect of ant secretions on pollen for all the analysed plant species except *Conospermum* species suggests that the investigated ants produce antimicrobial defences despite the dry summers that characterize south-western Australia. It is noteworthy that although the sugar ants *Camponotus molossus* and *Camponotus terebrans* do not possess a metapleural gland (Heterick, 2009), the detrimental effect on pollen grains in these two species was comparable to that of the meat ant *I. purpureus*, which, as with most ant species, possesses this gland. Similar outcomes were found for the pollen of *Cytinus hypocistis* after contact with the ant *Camponotus pilicornis* by De Vega *et al.* (2009). This adds to the idea that antibiotic secretions may be secreted from different glands and distributed throughout the cuticle in at least some ant species (Hull and Beattie, 1988).

The lipoidal secretions of ants are able to penetrate the pollen grain via a hydrophobic pathway and render the plasma membrane and the organelle membranes ineffective (Beattie *et al.*, 1985). The possible hydrophobic pathways are unknown, but it is plausible that *Conospermum* has a mechanism to mitigate the osmotic shock that leads to the lysis of the bilayer membrane of pollen. *Conospermum* pollen shows remarkably fast tube growth, orders of magnitude faster than with other plants. This may represent a possible difference in physiology that may be associated with its ability to cope with ant secretions. In fact, although pollen tube growth rate was not specifically investigated in this study, we noticed tube growth rates of the order of 50 µm s^−1^, in line with findings for other species in the genus *Conospermum*, including *C. amoenum*, *C. spectabile*, *C. eatoniae*, *C. caeruleum*, *C. brownii* and *C. incurvum*, where pollen tubes emerged and grew at rates of up to 55 µm s^−1^ (Stone *et al.*, 2004). These rates of pollen tube growth exceed some of the fastest recorded *in vivo* speeds, which were around 1.8 µm s^−1^ (evening primrose) to 2.7 µm s^−1^ (maize) (Stanley, 1971; Barnabas and Fridvalszky, 1984).

The effectiveness of a given pollinator depends not only on its abundance and floral visitation but also on the efficiency with which they collect and deposit pollen [De Vega *et al.*, 2009; and see Herrera (1987, 1989) for quantity and quality components of the plant–pollinator interaction, respectively). Ants are active floral visitors in the region and frequently visit our target species *C. undulatum*. Our results indicated that ants carried pollen of different plant species, but despite being generalist floral visitors, they presented a pollen load with a high proportion of *C. undulatum* grains. The characteristic pollination mechanism of *Conospermum* makes pollination by small insects unlikely. Indeed, we recorded many dipterans and small ants fatally trapped by the triggered style of *Conospermum*. However, all the species of ants we studied have workers larger than 7 mm in length, which allows them to forage within the calyx of *Conospermum* flowers untroubled by the trigger mechanism of the stigma. The stigma, once triggered, can easily reach the ant visitor’s body to collect the pollen deposited from previous floral visits to complete this characteristic pollination process.

Plant adaptation to cope with ant secretions and evidence of suitable pollen load carried by ants suggests that *C. undulatum* probably relies on both ants and the native bee *L. conospermi* for pollination. The contribution of ants to the reproductive output of this species was tested by means of exclusion treatments, and, in contrast to our initial hypothesis, we found that pollination by ants only (FLY_EX treatment) produced an unexpected 62.7 % of seeds compared to freely exposed controls; and ant-excluded plants resulted in significantly lower seed set than control plants available to both flying insects and ants. Thus, we demonstrated that pollination from winged visitors alone was not sufficient to allow *C. undulatum* to produce its maximum seed set in natural conditions, and therefore ants are probably playing an important role in filling this gap in the pollination of the species. Studies of many plant–ant interaction systems observed an increased occurrence of geitonogamous selfing (i.e. transfer of pollen between different flowers of the same plant) following ant pollination due to the restricted foraging area exhibited by the investigated ants that led them to repeatedly visit individual flowers in close proximity (e.g. Peakall and Beattie, 1991; Gómez and Zamora, 1992; De Vega *et al.*, 2009). However, in a recent study Delnevo *et al.* (2019b) found that *C. undulatum* possesses a strongly developed self-incompatibility system that prevents the development of the embryo following both autogamous and geitonogamous selfing. This suggests that, although the species lacks the reproductive assurance of self-compatibility, ant pollination produced outcrossed progeny and did not contribute to the often-negative effects of selfing on plants (Herlihy and Eckert, 2002). Moreover, the discrepancy between the sum of ANT_EX and FLY_EX treatments and the controls (i.e. the sum of the exclusion treatments exceeds 100 %) may be explained by the possible negative effect of introduced honeybees on the reproductive success of *C. undulatum*. Honeybees occur at high densities in the region due to the presence of domestic hives, and were recorded visiting *C. undulatum* flowers. However, *A. mellifera* is too big to pollinate the small flowers of *Conospermum*, and trigger the stigma with only their proboscis while foraging for nectar without inserting their head into the calyx; therefore, the stigma is unable to reach the body of the visitor to collect the pollen deposited during previous floral visits. Because the flowers of *Conospermum* can only be triggered once, this behaviour possibly decreases the relative contribution of ants to the reproductive output of freely exposed plants by reducing the availability of flowers to true pollinators, and probably increases pollen limitation. The impact of *A. mellifera* robbing nectar and pollen, and, in the case of *C. undulatum*, triggering the stigma without pollinating the flower, may have cascading negative effects on the reproductive success of native plants that coevolved with native pollinators to develop characteristic flower morphologies over long timeframes. This may be particularly important for threatened species such as *C. undulatum* and is worthy of further investigation.

Ants have been traditionally considered nectar thieves, and some plants are known to produce volatiles that repel ants (Willmer *et al.*, 2009). However, we have shown that mutualistic services by hymenopterans of the family Formicidae are important for maximizing the seed output in *C. undulatum*, together with the native bee *L. conospermi*. This adds to the growing body of research highlighting the important role of ants in some plant–pollinator systems (Sugiura *et al.*, 2006; De Vega *et al.*, 2009; Del-Claro *et al.*, 2019). Nonetheless, there is a scarcity of experimental evidence on the adaptation of plant species to cope with the usually detrimental ant microbial secretions. In many ant pollination studies it is unclear whether the ants produced less harmful secretions or whether the plants were adapted to cope with such secretions. In a recent study, De Vega *et al.* (2014) found evidence of adaptation by production of volatiles to attract ants in Mediterranean *Cytinus* species (Cytinaceae). However, pollen germination was negatively affected after contact with two species of ants (De Vega *et al.*, 2009), suggesting possible adaptation of some ant species to the Mediterranean climate of south-west Spain rather than pollen resistance, which contrasts with our finding for *Conospermum*. This highlights the complexity of ant–flower interactions and reinforces the fact that our understanding of these systems is still in its infancy.

*Conospermum undulatum* does not possess features of the proposed ‘ant-pollination syndrome’ (Hickman, 1974), such as small open flowers with a small amount of pollen and readily accessible nectaries, although this is also the case in a few other ant-pollinated plants (e.g. Peakall and Beattie, 1991; De Vega *et al.*, 2009). Therefore, it seems that *C. undulatum* has coevolved to facilitate pollination by *L. conospermi*, although coevolution also with native ants cannot be excluded.

Our study demonstrating the importance of ant pollination in this threatened species adds to the ecological roles that ants might play in the region, and the fact that ants produce antimicrobial secretions in this environment characterized by a Mediterranean climate do not preclude ant pollination in the Australian kwongan. Instead, our results indicate that such mutualistic associations can occur in unexpected ways, and open the way for future studies to investigate flower–ant interactions in this global biodiversity hotspot. Studies on *Conospermum*, as well as phylogenetically related taxa, will provide an opportunity for understanding where and when this trait evolved and how common it is amongst the flora of south-western Australia.
